# Cerumen microbial community shifts between healthy and otitis affected dogs

**DOI:** 10.1371/journal.pone.0241447

**Published:** 2020-11-25

**Authors:** Giorgia Borriello, Rubina Paradiso, Carlotta Catozzi, Roberta Brunetti, Paola Roccabianca, Marita Georgia Riccardi, Bianca Cecere, Cristina Lecchi, Giovanna Fusco, Fabrizio Ceciliani, Giorgio Galiero

**Affiliations:** 1 Istituto Zooprofilattico Sperimentale del Mezzogiorno, Portici, Italy; 2 Dipartimento di Medicina Veterinaria, Università di Milano, Milano, Italy; University of Lincoln, UNITED KINGDOM

## Abstract

Otitis externa is a common multifactorial disease in dogs, characterized by broad and complex modifications of the ear microbiota. The goal of our study was to describe the ear cerumen microbiota of healthy dogs, within the same animal and between different animals, and to compare the cerumen microbiota of otitis affected dogs with that of healthy animals. The present study included 26 healthy dogs, 16 animals affected by bilateral otitis externa and 4 animals affected by monolateral otitis externa. For each animal cerumen samples from the right and left ear were separately collected with sterile swabs, and processed for DNA extraction and PCR amplification of the 16S rRNA gene. Amplicon libraries were sequenced using an Ion Torrent Personal Genome Machine (PGM), and taxonomical assignment and clustering were performed using QIIME 2 software. Our results indicate that the bacterial community of the cerumen in healthy dogs was characterized by extensive variability, with the most abundant phyla represented by Proteobacteria, Actinobacteria, Firmicutes, Bacteroidetes and Fusobacteria. The analysis of both alpha and beta diversity between pairs of left and right ear samples from the same dog within the group of affected animals displayed higher differences than between paired samples across healthy dogs. Moreover we observed reduced bacterial richness in the affected group as compared with controls and increased variability in population structure within otitis affected animals, often associated with the proliferation of a single bacterial taxon over the others. Moreover, *Staphylococcus* and *Pseudomonas* resulted to be the bacterial genera responsible for most distances between the two groups, in association with differences in the bacterial community structure. The cerumen microbiota in healthy dogs exhibits a complex bacterial population which undergoes significant modifications in otitis affected animals.

## Introduction

Otitis externa is a common disease of dogs and is defined as the inflammation of the external ear canal, including the ear pinna. Its development is due to primary factors, such as parasites, foreign bodies, hypersensitivity and allergic diseases [1–3]. However, anatomic and conformational factors such as pendulous ears, excessive hair, increased cerumen production and excessive moisture can act as predisposing causes, by altering the local environment and increasing the risk of development of otitis externa. Infectious agents have been historically classified as perpetuating factors, but have been recently re-classified as secondary factors, which can lead to pathology only in abnormal ear environment or in combination with other predisposing factors [1–3]. Few bacterial genera have been traditionally identified by cytology and culture as components of the normal microflora of the ear canal of healthy dogs, including *Staphylococcus* spp. and *Bacillus* spp. [4,5]. The increase in the number of these bacteria or the proliferation of other opportunistic pathogens in the ear may lead to or facilitate the development, aggravation and/or perpetuation of inflammation [2,4].

The development and implementation of culture-independent methods based on high-throughput sequencing technologies have profoundly changed the view of the microbial community living on the skin and different body sites in dogs as in humans, identifying a much more complex bacterial community than recognized in the past [6–12]. The skin microbiota is fundamental for skin physiology and immunity so that alterations in its composition can undermine the proper epidermal barrier function [13,14]. Limited information describing the host-microorganism interaction during otitis externa has been reported so far. Recent findings indicate that ear canal diseases in dogs as in humans are characterized by significant complex modifications of the microbiota responsible for dysbiosis [9,15]. Another cause of skin and ear microbiota alteration with subsequent dysbiosis and possible proliferation of opportunistic pathogens in canids and other mammal species can be represented by mite infection [16–18]. Atopic dogs without clinical otitis have been shown to exhibit consistent changes in otic microbiota [19]. Decreased bacterial richness and diversity have been found in dogs with otits externa, with a severe increase of *Staphylococcus* spp. [20,21], whereas a very recent study demontrated a correlation between bacterial and fungal populations of the external ear canal and otitis caused by *Pseudomonas* and *Malassezia* [21]. *Malassezia pachydermatis* has been reported as the dominant fungi in the ear skin of healthy dogs, while dogs with clinical otitis exhibited an overgrowth of *M*. *globosa* and *M*. *sympodialis* [22].

Characterization of canine otic microbiome is at its beginning. There is a general need for identification of the main organisms colonizing healthy versus otitis affected dogs, otitis being a common disease of dogs and representing a therapeutical challenge due to recurrent and chronic infections and the emergence of antibiotic resistance. A comprehensive understanding of the microbial community interactions and shifts in the microbiota between healthy and otitis states is essential for the application of effective treatments of ear infection based on prevention strategies, treatment of infection and inflammation as well as the determination of leading causes of otitis development.

The aims of this study were (i) to define the cerumen microbiota of the ear canal of healthy dogs, (ii) to characterize the cerumen microbiota of otitis affected dogs, and (iii) to compare the microbiota of the two groups to assess the changes occurring in the cerumen microbial community structure in otitis affected dogs.

## Materials and methods

### Ethical statement

The dogs included in the present study were recruited during routine veterinary procedures by the veterinary clinics participating in the study, under informed consent of the owners and out of the scope of Directive 2010/63/EU (art. 1.5.f “practices not likely to cause pain, suffering, distress or lasting harm equivalent to, or higher than, that caused by the introduction of a needle following good veterinary practice”). All applicable international, national, and/or institutional guidelines for the care and use of animals were followed. The study design was approved by the Italian Ministry of Health, project n. IZS ME 13_15 RC.

### Study population

The present study included 46 dogs, consisting of 26 healthy dogs recruited during routine clinical examination for vaccination or annual check-up visits, 16 dogs affected by bilateral otitis externa and 4 dogs affected by monolateral otitis externa. Informed owner consent was obtained before any procedures. The animal ID, breed, sex, age, and clinical diagnosis of all dogs included in this study are presented in the **S1 Table**. The same population, with the relative cytological data, was included in a previous study on microRNAs of cerumen from dogs affected by otitis externa [23].

Control subjects were classified as healthy based on lack of history and clinical signs of otic disease, physical and otoscopic examination and on the absence of inflammatory cells, bacteria <25 and yeast < 5 per high power microscopy field (400x) on ear cytological specimens. Clinical otitis externa was diagnosed based on clinical signs such as head shaking, pruritus, local pain, otorrhoea, erythema or swelling of at least one ear canal, visible debris and discharge in the ear canal upon otoscopic examination and cytological confirmation of bacterial infection by microscopical ear cerumen specimen examination. For microscopical examination, the ear cerumen was collected from the external ear canal with cotton-tipped swabs and then streaked onto two glass slides, which were then heat-fixed and stained with a modified Wright's stain (Quick Panoptic Kit; Pokler Italia). At least 10 fields per slide were examined under optical microscopy and a total number of bacteria (cocci or rods) ≥25 per high power microscopy field (400×) with or without bacterial phagocytosis by neutrophil granulocytes were considered positive (infection) as previously described [24]. Cases were excluded if they had received any topical ear treatment within the two weeks before sampling, or any systemic antifungal and/or antibiotic or any probiotic treatment during the three months before sampling. Control subjects were classified as healthy based on history, physical and otoscopic examination and on the absence of neutrophil granulocytes, bacteria <25 and yeast < 5 per high power microscopy field (400x) on ear cytological specimens.

### Sample collection and DNA extraction

Each animal included in this study was subjected to general anaesthesia. For each dog, the ear cerumen from the right and left ear were separately collected on sterile BD flocked swabs (BD Biosciences, NJ) by rubbing the skin of the horizontal cartilaginous portion of the ear canal, close to the tympanic membrane, for 10 seconds. Careful handling was applied to avoid sampling of the ear pinna skin and the outermost portion of the vertical ear canal. A total of 92 swabs (52 from 26 healthy dogs, 8 from 4 dogs affected by monolateral otitis externa and 32 from 16 dogs affected by bilateral otitis externa) were collected. Each sample was labeled and stored at -80°C until analysis. DNA was extracted by the DNeasyPowerSoil Kit (MO BIO Laboratories, Inc) according to the manufacturer’s instructions, including also negative extraction controls. DNA was quantified using a high-sensitivity Qubit^TM^ fluorometer.

### Amplification and sequencing

The 16S rRNA gene was amplified by the 16S Ion Metagenomics kit (Life Technologies) following the manufacturer’s instructions. Briefly, two separate PCR reactions were carried out, amplifying respectively V2-4-8 and V3-6, V7-9 regions. Each PCR reaction included 5 ng of microbial DNA and was characterized by a thermal profile consisting of the following steps: 1 cycle at 95°C for 10 min, 30 cycles consisting of 95°C for 30 sec, 58°C for 30 sec, 72°C for 20 sec, and a final extension cycle at 72°C for 7 min. After amplification, PCR products were purified using the Agencourt AMPure beads (Beckman Coulter Inc, Atlanta, Georgia), eluted in Low TE buffer and quantified by the Qubit dsDNA HS Assay kit (Life Technologies). Reactions included PCR and extraction of negative controls to evaluate the effect of possible contamination along the processing workflow [25–27]. For library preparation, for each sample, 50 ng of DNA from each PCR reaction were pooled to have 100ng of total DNA to be used for further processing. Libraries were barcoded using Ion Xpress Barcodes Adapters (Life Technologies) and amplified in an emulsion PCR on the One-Touch 2 and One-Touch ES systems (Life Technologies) according to the manufacturer’s instructions. Sequencing was performed on the Ion Personal Genome Machine (PGM) using the Ion 318 Chip kit V2 (Life Technologies). The raw sequences have been submitted to NCBI under BioProject accession number PRJNA649402.

### Data analysis

After sequencing, reads pre-processing for quality control was performed by DADA2 to denoise, remove primers, de-replicate single-end sequences, remove chimaeras and exclude low quality reads [28,29]. Based on the quality control check, filtered and de-noised reads were trimmed at 250 bases and resolved to high-resolution Amplicon Sequence Variants (ASVs), which represent, as closely as possible, the original biological sequence of the sequenced amplicon [29]. The downstream taxonomic analysis at phylum, family and genus level was carried out by the QIIME 2–2020.2 software. For this purpose multiple sequence alignment of representative ASVs sequences was carried out using MAFFT software [30]. FastTree [31] software was then used to infer unrooted and subsequently rooted maximum-likelihood phylogenetic trees representing the phylogenetic relatedness of ASVs (QIIME2 phylogeny align-to-tree-mafft-fasttree plugin). ASVs were taxonomically classified using a Naïve Bayes classifier, pre-trained on GreenGenes v.13-8 reference sequences clustered at 99% similarity (QIIME2 feature-classifier classify-sklearn plugin) [32,33]. Samples characterized by less than 9000 sequences were excluded from the analysis. Chloroplasts and not classified sequences were also excluded, given that the total number of found sequences was negligible. Of the 92 cerumen samples, one sample belonging to healthy ears (ID #18 right ear) and two samples belonging to otitis affected ears (#41 left ear and #42 left ear) exhibited a limited number of sequences (less than 9000) and were therefore excluded from the study. After reads pre-processing of the 89 ear samples included in the study, the total number of sequences was 3,296,371, with a mean number of sequences of 42,277 per sample in affected ears (median number: 40,148 sequences, minimum number: 9,278 and maximum number: 91,938) and 33,744 in healthy ears (median number: 30,067, minimum number: 16,599 and maximum number: 72,878). Negative controls were sequenced and the identified features (**S2 Table**) were evaluated in order to exclude the presence of any contamination able to critically impact results [25–27]. All the features found in negative controls were absent in the cerumen samples. Based on this evidence and the best practices for analyzing microbiomes [25–27], and in consideration of similar studies in the literature [9,16], we decided to filter out low abundance ASVs by removing all the features with a minimum frequency lower than 150. Data were first evaluated by the Kolmogorov-Smirnov test to verify their non-normal distribution. Following taxonomic analysis, statistical differences of population abundance between groups were evaluated by the analysis of composition of microbes (ANCOM) and differential abundance analysis with gneiss balance. ANCOM calculates pairwise log-ratios between combinations of taxa and considers how many times (W) the null hypothesis (no differenxe between each pairwise comparisons of taxa) is violated [34], while gneiss calculates log-transformed ratios (balances) between groups of taxa arranged in a hierarchical tree thus identifying taxa that differ in relative abundance [35,36]. Both analysis require a filtering-out step in which taxonomic features occurring in fewer than 10 samples or with frequencies below 50 are removed in the input step. Significant balances were identified by the use of an ordinary least squares (OLS) regression model with health status as covariant using the ols regression command in QIIME2. Identification of specific features of interest was performed by BLAST analysis [37]. A specific plugin in QIIME 2 [38] (http://github.com/qiime2/q2-longitudinal) was used to analyse differences in alpha and beta diversity for paired samples (right and left ear collected from the same animal). This analysis excluded the group of subjects with monolateral otitis which was characterized by a too low number of samples. Alpha diversity analysis between healthy and affected dogs, based on Chao1 (a measure of species richness based on ASVs abundance) and Shannon (a quantitative measure of both the number of species and the inequality between species abundances) indexes was performed using the Mann-Whitney test for unpaired samples. Samples (healthy and bilateral otitis) were also analyzed for beta diversity Principal Coordinates Analyses (PCoA) using Bray Curtis (a quantitative measure of community dissimilarity), unweighted (a qualitative measure of community dissimilarity incorporating phylogenetic relationships between the features) and weighted (a quantitative measure of community dissimilarity incorporating phylogenetic relationships between the features) UniFrac distances matrices in the QIIME 2 software [39]. Groups dissimilarity was tested both by Permutational Multivariate Analysis of Variance (PERMANOVA) and Permutational Analysis of Multivariate Dispersions (PERMDISP). PERMANOVA is a non-parametric multivariate statistical test used to assess whether the centroids and dispersion of the groups are equivalent for all groups. The null hypothesis of this test is that the metric centroid does not differ between groups. PERMDISP is a multivariate test which evaluates the homogenity of dispersion within groups [40]. The null hypothesis of this test is that the average within-group dispersion is the same in all groups.

## Results

### Subjects

Forty-six dogs (26 healthy, 4 affected by monolateral otitis externa and 16 affected by bilateral chronic otitis externa) were included in the present study (**S1 Table**). The median ages of the two groups were 8.3 years (ranging from 0.5 to 13 years) for the healthy group and 7 years (from 1 to 14 years) for the otitis affected group. The male-to-female ratio was 0.67 in the control group and 1.12 in the otitis group. The healthy group included 16 different breeds, 3 mixed breeds and 4 not reported breed, while the otitis group included 17 different breeds, 2 mixed breeds and 1 not reported breed. For each dog, swabs from both ears were collected for a total of 92 samples.

### Cerumen microbiota in healthy dogs

**Fig 1:**
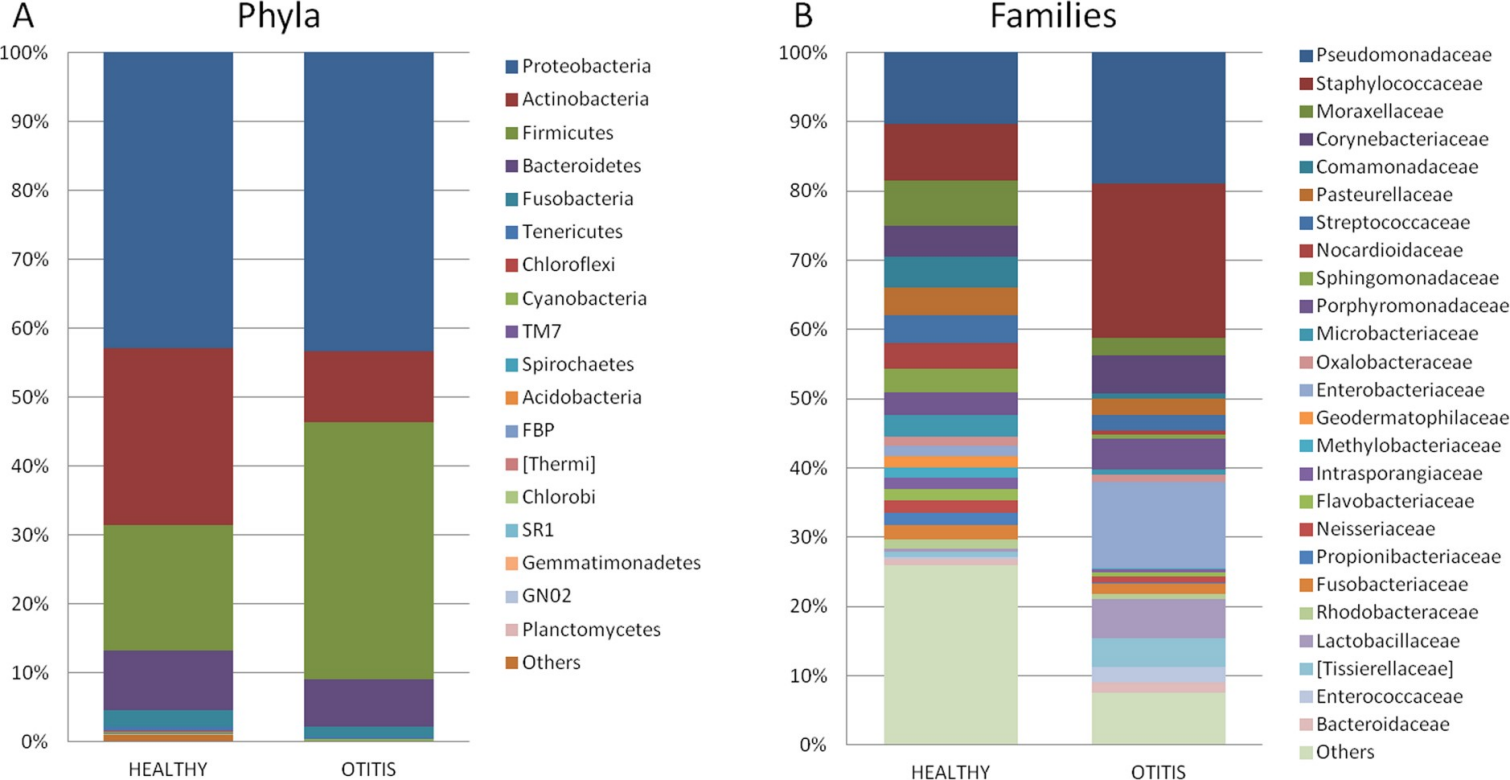
Taxonomy bar plot at Phylum and family level of cerumen samples from healthy and otitis affected dogs. Relative abundance of the bacterial taxa identified in cerumen samples from healthy and affected dogs at different taxonomic levels: (A) Phyla: reported values represent the mean relative frequency for each Phylum calculated in the healthy (n = 55) and otitis (n = 34) group, respectively; (B): Family: reported values represent the mean relative frequency of each Family (with a relative frequency >1% in at least one group) for each group.

The analysis of 55 cerumen samples from healthy ears exhibited the presence of 19 phyla (**Fig 1A**). The most abundant (with mean relative frequencies >2%) were Proteobacteria (mean frequency value of 42.9% ± standard deviation of 17.2%), Actinobacteria (25.6%±16.4%), Firmicutes (18.3%±17.1%), Bacteroidetes (8.7%±7.8%) and Fusobacteria (2.3±5.8). At the family level (**Fig 1B**), the most abundant were *Pseudomonadaceae* (10.1%±14.2%) followed by *Staphylococcaceae* (8.0±16.8%%), *Moraxellaceae* (6.4%±6.2%), *Corynebacteriaceae* (4.4%±12.0%), and *Comamonadaceae* (4.2%±6.9%). Finally, at the Genus level, a total of 391 genera were observed in the normal ear, with the most frequent (more than 2%) represented by *Pseudomonas* (9.8%±14.2%), *Staphylococcus* (7.6%±16.8%), *Corynebacterium* (4.3%±12.0%), *Streptococcus* (3.7%±4.3%), *Porphyromonas* (3.1%±4.7%), *Acinetobacter* (2.9%±2.6%), an unclassified genus of the family *Pasteurellaceae* (2.8%±7.0%), *Sphingomonas* (2.4%±3.4%) and an unclassified genus of the family *Nocardioidaceae* (2.3%±2.3%).

### Cerumen microbiota in otitis externa dogs

The analysis of 34 cerumen samples from otitis externa dogs exhibited the presence of 18 phyla (**Fig 1A**). The most abundant (with mean relative frequencies >2%) were Proteobacteria (43.3%±34.4%), Firmicutes (37.2%±33.3%), Actinobacteria (10.4%±14.8%), and Bacteroidetes (6.9%±12.2%),. At the family level (**Fig 1B**) the most abundant were *Staphylococcaceae* (22.2%±31.9%), *Pseudomonadaceae* (18.9%±22.7%), *Enterobacteriaceae* (12.4%±20.0%), *Lactobacillaceae* (5.5%±17.8%), *Corynebacteriaceae* (5.4%±11.5%), *Porphyromonadaceae* (4.5%±9.6%), and *[Tissierellaceae]* (4.1%±8.6%). Finally, at the Genus level, a total of 316 genera were observed in the otitis externa affected ears, with the most frequent (more than 2%) represented by *Staphylococcus* (22.0%±32.0%), *Pseudomonas* (18.6%±22.8%), *Proteus* (5.6%±11.3%), *Lactobacillus* (5.5%±17.8%), *Corynebacterium* (5.4%±11.5%), an unclassified genus of the family *Enterobacteriaceae* (4.9%±10.5%), *Porphyromonas* (4.5%±9.6%), *Enterococcus* (2.3%±4.8%) and *Streptococcus* (2.2%±5.0%).

### Comparison between cerumen microbiota in healthy dogs vs. otitis externa dogs

**Fig 2:**
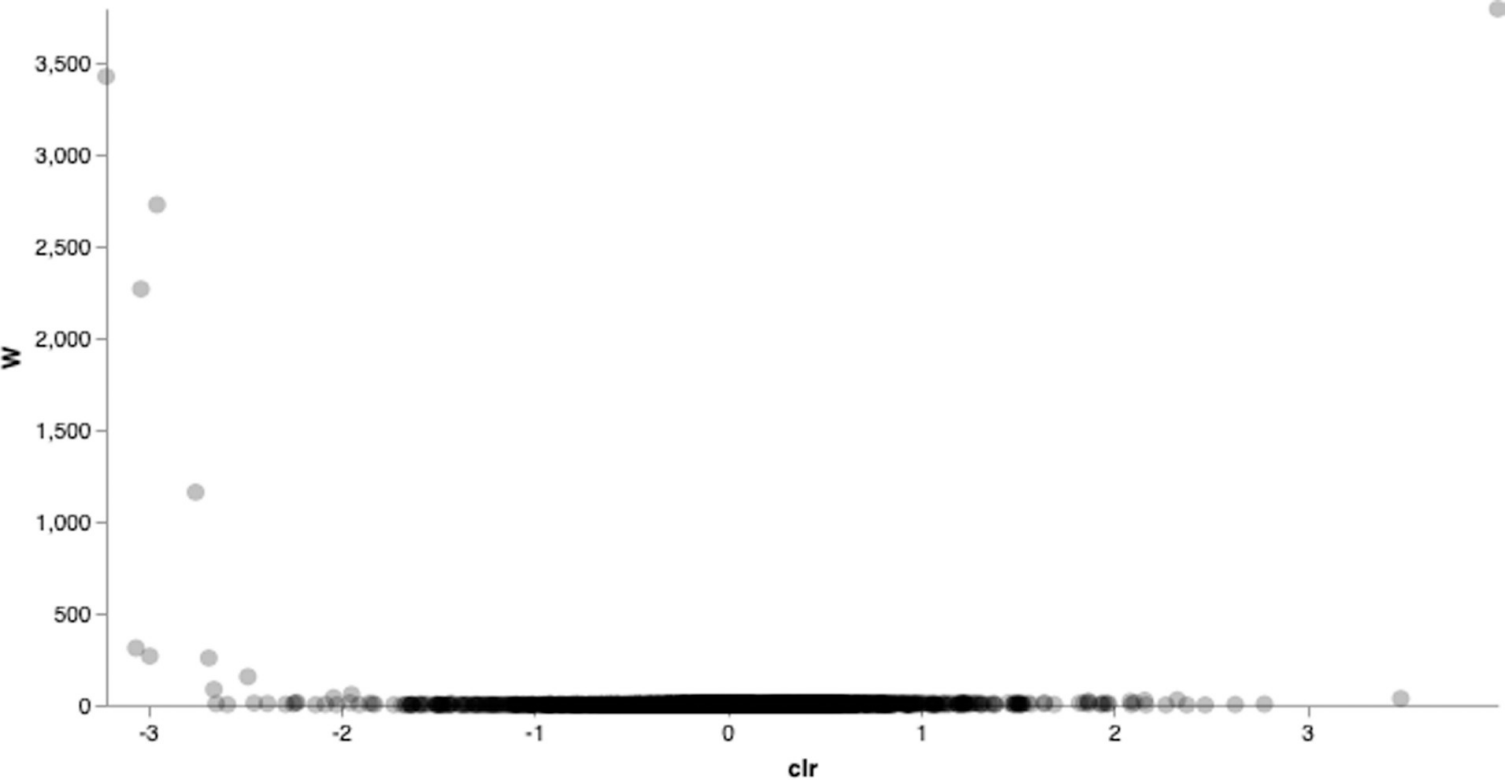
Taxa differentially abundant between healthy and affected dogs. ANCOM results identified two features as differentially abundant between groups. Groups included only one cerumen sample for each animal (right ear for each animal).

The taxonomic composition and relative abundances of cerumen samples from healthy and affected animals were analysed by both the analysis of composition of microbes (ANCOM) and gneiss balance to identify differentially abundant bacterial taxa between healthy and otitis affected dogs. ANCOM analysis restituted 17 features as differentially abundant between healthy and affected dogs (**S1 Fig**). Among these features, 11 ASVs were identified by BLAST analysis as *Pseudomonas* spp. (W = 5025; W = 5019; W = 4997; W = 4991; W = 4893; W = 4888; W = 4861; W = 4845; W = 4810; W = 4688; W = 4625, respectively), one ASV as *Staphylococcus* spp. *(*W = 4965*)*, one ASV as *Sphingomonas* spp. (W = 4897), two ASVs as *Proteus* spp. (W = 4892; W = 4650, respectively), one ASV as a member of the family *Methylobacteriaceae* (W = 4771), and one ASV as *Propionibacterium acnes* (W = 4716), respectively (**S3 Table**). Since our sample size included cerumen samples from both ears of the same animal, in order to confirm the reliability of our data by excluding pseudoreplication problems, we carried out the same ANCOM analysis on a sub-set of samples, including only one cerumen sample for each animal (**Fig 2**). ANCOM results confirmed the features 34122cc129525e668e0b4c8f56a42558 (W = 3789) and c961a6807004cea4e0c552f07b4f8450 (W = 3421), as differentially expressed between groups, represented by the two outliers in the volcano plot (**Fig 2)**. These features were identified by BLAST analysis as *Staphylococcus* spp. and *Pseudomonas* spp., respectively (**S4 Table**). Three more features were present in the volcano plot characterized by a W value >1000, e95cc3c507eb14495dda55e98d378a2d (W = 2723), eaeec474201946244a2bc88b3effd5ae (W = 2264) and 54e996c8921772176f4dbc08c1e6b535 (W = 1158), which were identified by BLAST analysis as *Pseudomonas* spp., *Pseudomonas* spp. and *Moraxella* spp., respectively. The latter ASV was present only in two affected dogs, while was more abundant in the healthy group (16 animals). Gneiss analysis allows to infer changes in the balance between particular subsets of the microbial community across different covariates. Balances can be evaluated using bifurcating trees which can be built relating microbial taxa to each other by using a desired criterion. Balances toward the tips of the tree have been shown not to impact each other, and can therefore be ignored, while attention should be focused on the balances closer to the root of the tree, which are those containing most information and have the potential to explain large shifts in the microbial community under study [35,36]. Hierarchical clustering of ASVs by health status followed by OLS regression analysis exhibited a linear regression model fit of 0.06, indicating that health status accounted for 6% of the variation. Log ratio balances statistically significant with respect to otitis were y_0_ (β = -13.7; p<0.0001), y_2_ (β = -9.8; p<0.05), y_8_ (β = 1.4; p<0.05) and y_12_ (β = -2.1; p<0.05) (**S2 Fig**). These balances indicated an overall increase of *Pseudomonas*, *Proteus* and *Staphylococcus* in otitis affected dogs. The same analysis carried out on the sub-set of samples including only one cerumen sample for each animal exhibited an increase in the R2 value, indicating that otitis, in this sample population, accounted for 13.6% of the observed variation, and four significant balances, y_0_ (β = -8.4; p<0.005), y_5_ (β = 2.4; p<0.005), y_6_ (β = 0.34; p<0.05) and y_8_ (β = -0.95; p<0.05) (**S3 Fig**). These balances confirmed the increase of *Pseudomonas* and *Staphylococcus* in otitis affected dogs, and also showed an increase of *Streptococcus*, *Corynebacterium* and *Enterococcus* in the same group.

**Fig 3:**
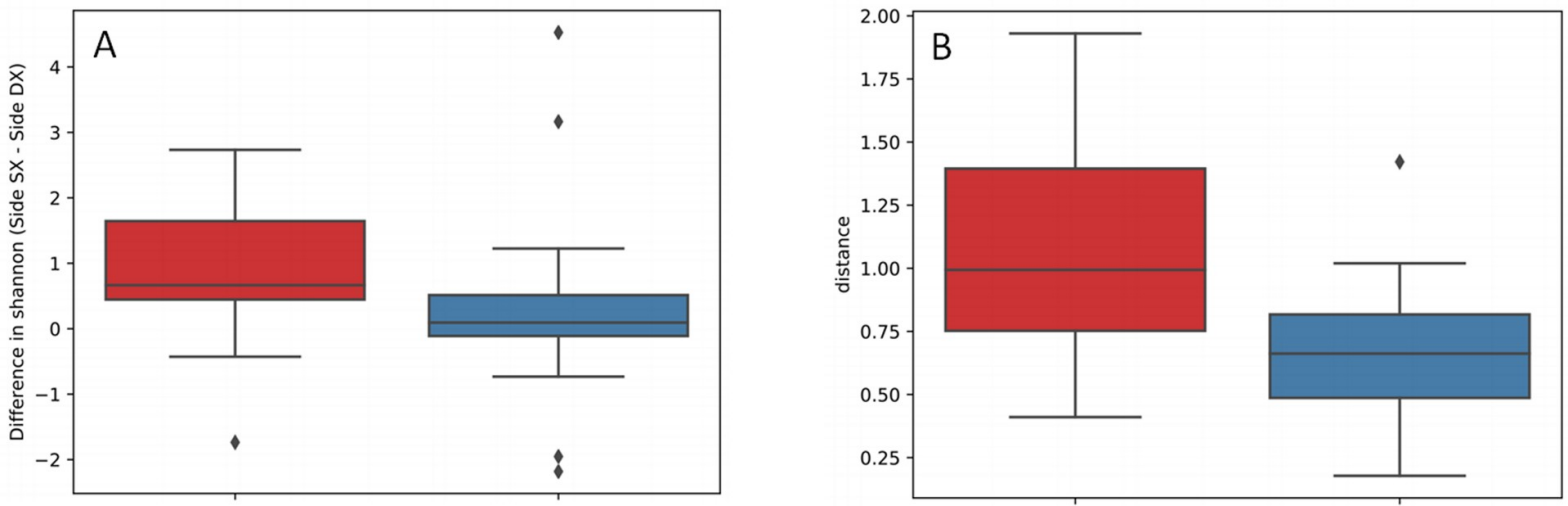
Apha and beta diversity analysis between the two ears from the same animal. Paired differences and distances between right and left ear from the same animal in healthy and otitis affected dogs: (A) Shannon index (P = 0.057); (B) weighted UniFrac matrix (P = 0.021).

A specific analysis was performed on paired samples (right and left ear from the same animal) to evaluate differences across samples for alpha and beta diversity. Results indicated that while differences in microbial richness (Kruskal-Wallis test; Chao1 index; *H* = 0.161; *P* value *=* 0.697*)* and evenness (Kruskal-Wallis test; Shannon index; *H* = 3.621; *P* value *=* 0.057*)* were not significant, distances based on both presence and abundance (Kruskal-Wallis test; weighted UniFrac matrix; *H* = 5.349; *P* value *=* 0.021) (**Fig 3**) between right and left ears from the same dog across affected animals were higher than in healthy subjects, demonstrating that the microbiota structure of cerumen between right and left ear exhibits greater changes across affected animals than in healthy ones.

**Fig 4:**
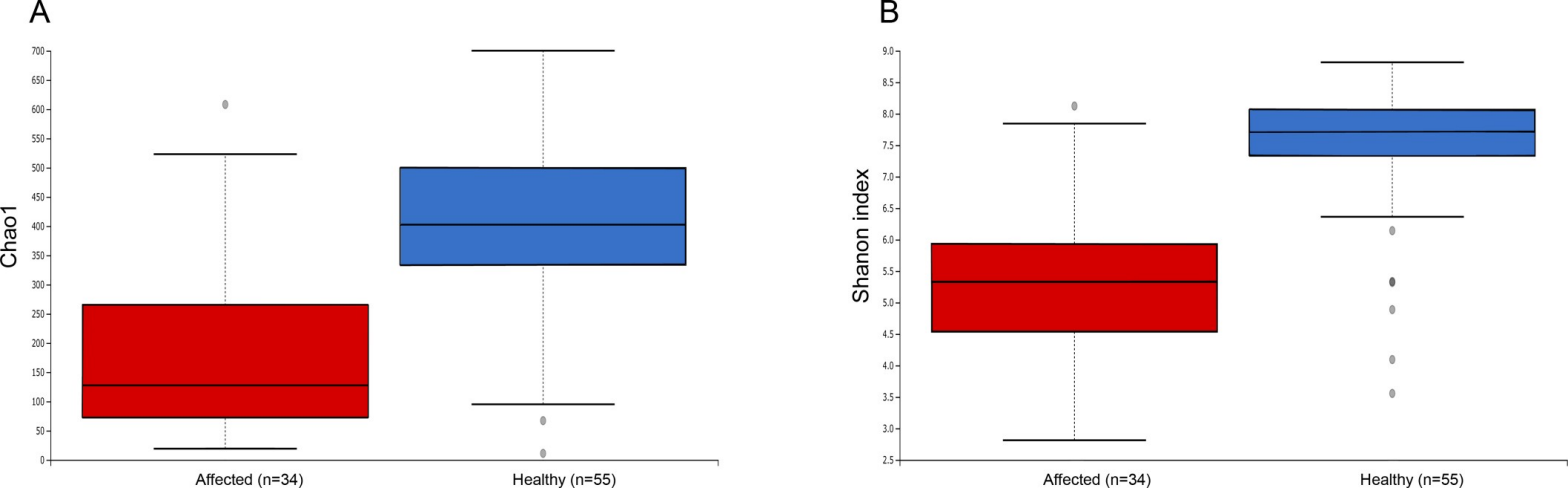
Alpha diversity analysis between healthy and affected dogs. Differences in alpha diversity metrics between healthy and affected dogs: (A) Chao1 index; (B) Shannon index.

The comparison of the observed ASVs number between healthy and affected dogs revealed that most samples in the control group exhibited a high number of observed ASVs (mean ASVs number 408 ± standard deviation 133), while among affected dogs, most samples exhibited a lower number of observed ASVs (mean ASVs number 185 ± standard deviation 138). Alpha diversity analysis displayed significant differences between healthy and affected dogs both in the microbial richness (Kruskal-Wallis test; Chao1 index; *H* = 32.266; *P* value < 0.001) and evenness (Kruskal-Wallis test; Shannon index; *H* = 31.247; *P* value < 0.001), indicating that the cerumen microbiota in healthy dogs was characterized by a higher number of bacterial taxa more evenly distributed than in affected dogs (**Fig 4A and 4B**). The same alpha diversity analysis carried out on the sub-set of samples including only one cerumen sample for each animal (**S4 Fig**) confirmed the presence of statistically significant differences between the two groups (Kruskal-Wallis test; Chao1 index; *H* = 13.625; *P* value < 0.001; Shannon index; *H* = 13.336; *P* value < 0.001).

**Fig 5:**
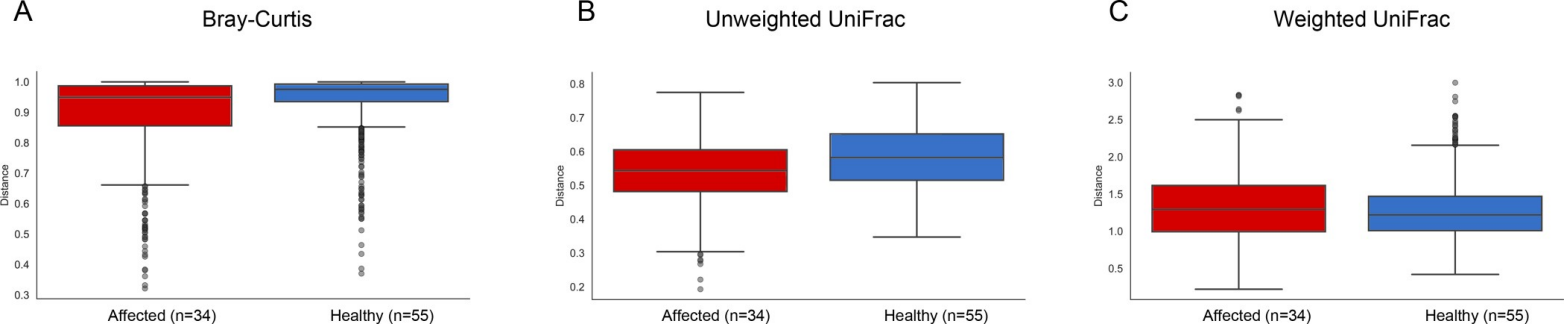
Beta diversity analysis between healthy and affected dogs. Bray Curtis (A), Unweighted (B) and Weighted UniFrac (C) distance matrices present an intra-group variation in healthy and affected dogs.

The beta diversity of the microbiota of the two groups was evaluated comparing the intra-group distances by both taxonomic and phylogenetic approaches. Measures of species abundance (PERMANOVA; Bray-Curtis; *pseudo-F =* 5.951; P = 0.001; **Fig 5A**), presence (PERMANOVA; unweighted UniFrac; *pseudo-F =* 12.156; P = 0.001; **Fig 5B**) and both presence and abundance considered together (PERMANOVA; weighted UniFrac; *pseudo-F =* 9.675; P = 0.001; **Fig 5C**) showed that the diversity within healthy ears was characterized by significantly less microbial variability than that within the affected ones. The Permutational Analyses of Multivariate Dispersions (PERMDISP) indicated that there was heterogeneity in multivariate dispersion within groups, for both Unweighted and Weigheted UniFrac matrix (PERMDISP; Bray-Curtis; *F-value =* 0.0023; P = 0.96; Unweighted UniFrac; *F-value =* 8.560; P = 0.005; Weighted UniFrac; *F-value =* 25.049; P = 0.001). An influence on samples homogeneity can be partly attributed to different sample size between groups. Moreover, as our sample population included cerumen collected from dogs of different age and sex, we performed significance testing for alpha and beta diversity to determine whether any of these variables had a significant effect on microbiota structure and samples homogeneity (**S5 Table**). Among tested variables, the breed was not considered since many groups along the grouping vector included only a single sample and was therefore considered as not representative (PERMDISP analysis not allowed). Results indicated that sex and age variables were homogeneously distributed within groups and were not associated with significant differences in cerumen microbiota structure between groups. Otitis appeared therefore, the main driving factor explaining the observed differences in alpha and beta diversity between groups.

Significant differences in Bray-Curtis dissimilarity index (PERMANOVA; *pseudo-F* = 3.905; P = 0.001), unweighted (PERMANOVA; *pseudo-F =* 7.055; P = 0.001) and weighted (PERMANOVA; *pseudo-F =* 2.725; P = 0.032) UniFrac distance matrices were still observed when the same analysis was carried out on the sub-set of samples including only one cerumen sample for each animal (**S5 Fig**). Interestingly, samples in this sub-set displayed a homogeneous dispersion (PERMDISP; Bray-Curtis dissimilarity index; *F-value* = 0.959; P = 0.340; UnWeighted UniFrac; *F-value* = 0.495; P = 0.513; Weighted UniFrac; *F-value* = 0.006; P = 0.948) thus supporting the hypothesis of otitis as the principal factor shaping alpha and beta diversity in the cerumen microbiota.

**Fig 6:**
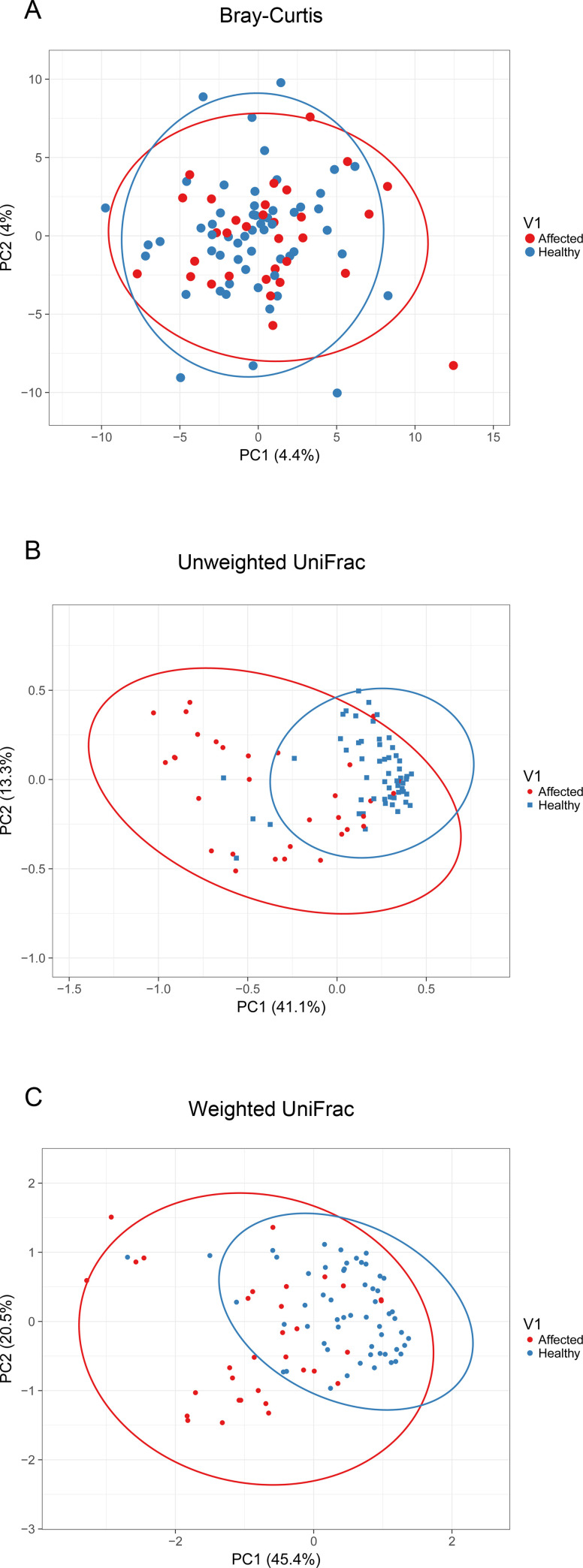
Beta diversity principal coordinates analysis between healthy and affected dogs. PCoA analysis based on Bray Curtis (A), Unweighted (B) and Weighted UniFrac (C) distance matrix between healthy and affected dogs.

Samples were subjected to PCoA analysis to evaluate the variability of microbiota structure between healthy and otitis externa affected dogs. Results indicated a significant difference (P = 0.001) between the two groups for all the three considered matrices (**Fig 6**), and, for both Unweighted and Weighted UniFrac distance matrix, samples clustered mostly along PC1, which could explain 41.1–45.4% of the variation.

Finally, the microbial diversity at the genus level based on assigned taxa, displayed that most samples from healthy dogs were characterized by a high number of genera with no predominance of a bacterial genus over the others, while among affected dogs, most samples exhibited a lower number of observed genera with the presence of at least one bacterial genus at a frequency value higher than 10%, either alone or in association with another genus. The latter category included 13 samples characterized by a high frequency of *Staphylococcus* (ranging from 14% to 99%), 18 samples exhibiting abundant amounts of *Pseudomonas* (from 11% to 81%) and 7 samples exhibiting a considerable presence of *Proteus* (from 11% to 42%).

## Discussion

This study describes the differences in microbial composition and diversity in the cerumen collected at the innermost part of external ear canal both within the right and left ear of the same animal and between healthy and otitis affected dogs. Our results indicate that the bacterial community of the normal ear canal is characterized by extensive variability. Otitis affected dogs show higher changes in cerumen bacterial population structure between left and right ear of the same animal, with respect to healthy dogs. Moreover we observed reduced bacterial richness in the affected group as compared with controls and increased variability in population structure within otitis affected animals, with the genera S*taphylococcus* and *Pseudomonas* acting as the main bacterial taxa responsible for the difference observed between the two groups. All these results were confirmed by carrying out the same analyses in a sub-set of samples including only one cerumen sample for each animal in order to exclude possible confusing results due to pseudoreplication.

Pseudoreplication is defined as the use of inferential statistics where replicates are not statistically independent [41,42]. Indeed psuedoreplication risks to inflate the evidence supporting a scientific claim by artificially enlarging the sample size and contributes to irreproducible results [42]. Since for each animal we collected cerumen samples from both ears, we decided to carry out two parallel analyses, one on the entire sample size (n = 89), and the other one on a sub-set of samples including only one cerumen sample (from the right ear) for each animal (n = 42). All the results obtained by the first analysis were confirmed by the second one, therefore supporting the reliability of our data.

The microbial diversity found in the ear canal in this study is consistent with other previous findings based on 16S rRNA amplicon sequencing [7,12,15,19–21]. The application of high throughput sequencing methods to ear microbiota analysis evidenced that the ear bacterial community in dogs is much more complex than known in the past based on culture-dependent methods [7,12,15,19–21]. Our results showed the presence of bacteria belonging to 19 different Phyla in healthy dogs, a value that is consistent with those reported in previous studies [19,20]. A similar richness in microbial composition has been also reported both in the ear and several body sites in canine [7,12] and other mammalian species, such as red foxes, grey foxes, coyotes, swine [16–18], including man [8,10,11,43]. The characterization of host-associated microbiomes is of pivotal importance in the field of biological studies thanks to the functional role played by resident bacteria on the physiology and immunity of the specific body site. The study of the taxonomic composition and diversity can provide useful information not only on the overall membership of the population, but also on the distribution of those members within the microbiota of different body sites and/or different host species. Perturbations of the skin microbiota are often associated with skin disorders and molecular studies can help to understand the microbial contribution to the disease and at the same time allow the identification of novel pathogens and/or protective bacteria [44].

In this study, we compared the cerumen microbiota of the two ears from the same animal. The analysis of paired samples can provide valuable information about subject heterogeneity [45] allowing to determine whether microbial diversity significantly changes between two different body sites. Alpha and beta diversity analysis indicated that paired samples (ears from the same animal) showed higher changes in cerumen bacterial population richness and structure across affected animals than in healthy ones. These data suggest that the otitis state is associated with a dysbiosis characterized by low consistency between ears in the same animal. These findings are coherent with previous reports [20] where beta diversity demonstrated that, in the group of healthy animals, samples from the same dog were no more similar to each other than samples from other healthy dogs. In contrast, the same study reported that the beta diversity analysis carried out in the otitis group showed that samples coming from the same animal clustered closely together and were more similar to each other than samples coming from other affected dogs, which is partially in disagreement with the current study. The difference is explained by the use of different metrics and statistical tests, as we used a specific QIIME tool for paired samples and applied it to the weighted UniFrac metric which considers both abundance and phylogenetic information, while Korbelik and colleagues considered the Jaccard index which accounts for presence/absence of taxa. Additionally, the cutaneous microbiota has been reported to be affected by individual and environmental factors [7,10] which might be also responsible for different results on different samples. This is why multiple studies may concur to provide a deeper understanding of possible differences in the otic microbiota in dogs.

Regarding microbiota in dogs with otitis, our results are consistent with those of previous reports [7,10,19,21], showing a reduced bacterial richness in otitis affected dogs. Indeed, in the otitis affected group, we found 18 phyla and a total of 316 genera vs. the 19 phyla and 391 genera detected in healthy animals. These reduced numbers closely parallel those reported in previous reports [19,20]. A reduced number in bacterial taxa might contribute to minor differences in beta matrices, as reported by Korbelik and colleagues [20]. The changes observed in the microbiota between healthy and affected animals indicate significant differences both in the overall composition of the bacterial population and in the distribution of the most represented taxa. The main phyla in both groups are unchanged, although with different relative abundances between healthy and affected animals. As indicated by the taxonomic analysis, the main bacterial genera accounting for most of the distances between healthy and affected dogs are *Staphylococcus* and *Pseudomonas*, which are more abundant in the latter group and are the pathogens most commonly associated with otitis externa in dogs [3,4,46]. Our results indicated that these two genera were the most abundant both in healthy and affected animals, but with different relative proportions. While the presence of *Staphylococcus* spp. has been widely demonstrated in the ear microbiota of healthy dogs [19–21], *Pseudomonas* spp. has been elsewhere found at lower frequencies in healthy animals [20]. Anyway this bacterial genus includes ubiquitous bacteria often found on the skin and ear microbiota of animals and humans [19–21,43], and this evidence might support the hypothesis that dysbiosis or use of antibiotics can select the flora to a purulent otitis with multi resistant *Pseudomonas*. Moreover, at the genus level, the microbiota of most affected animals appeared to be characterized by high relative abundance (>10%) of a single bacterial genus, either alone or in combination with another one. In particular, the genera *Staphylococcus* and *Pseudomonas* exhibit very high frequencies (up to 99% and 81%, respectively) in some affected animals, while, in the same group, other genera, such as *Proteus*, although showing high relative abundances, do not exceed values of 42%. Interestingly, in a similar study conducted on a referral population of dogs in the eastern USA, the reduction of bacterial diversity is more evident in dogs where the culture-based methods identified only cocci (≥25/high power field) rather than only rods [21]. The over-representation of a genus over the others is consistent with the reduction of bacterial variability in affected animals found both here and in analogous studies [7,10,19,21]. Whether these changes in bacterial genera proportions are due to the active proliferation of the pathogen over the natural microflora or to the depletion of the latter in favour of the increase of a single genus is still unknown. The present data suggest that there are differences in bacterial community structure associated with the specific bacterial genus implicated in otitis development and perpetuation, which might reflect differences in pathogenesis mechanisms, microbial community interactions and host-microbe interactions. However further studies will be required to address this topic.

Among the most abundant pathogens in the otitis affected group, besides *Staphylococcus* and *Pseudomonas*, the genera *Corynebacterium*, *Proteus*, *Lactobacillus*, *Streptococcus*, *Porphyromonas* and *Enterococcus* resulted significantly over-represented than in healthy dogs. In a previous study [47] *Corynebacterium* has been reported as positively correlated with staphylococcal infections in dogs with atopic dermatitis, even though the same authors, in another study carried out on otitis affected dogs characterized by either high cocci or high rods isolation, report that this pathogen was significantly decreased only in the cocci group and not in the rods one [21]. Indeed, the role of this pathogen in otitis development is controversial, and some authors suggest that it requires the presence of other bacteria to proliferate [48]. Moreover, in another study on mange-infected canids, *Corynebacterium* has been found associated with *Staphylococcus* and *Streptococcus* in infected animals [17]. *Proteus*, *Streptococcus* and *Enterococcus* have been widely associated with canine otitis externa [3,4] even though other studies on affected dogs did not identify a significant number of sequences attributable to either *Proteus* or *Enterococcus* by metagenomic analysis [19,21]. These differences might be related to several factors including the sampling of deeper portions of the ear and sequencing of different hypervariable regions of the 16S rRNA gene, which are known to impact microbiome results since they include different species-specific sequences and exhibit different degrees of sequence diversity [49]. Also, according to previous findings [21], our results do not show a significant presence of *Escherichia* either in the control or the affected dogs, even though the presence of an unclassified genus of the *Enterobacteriaceae* family has been detected in both groups, while another study [19] identified this genus as a common member of the microbiota of the ear canal of healthy dogs. As discussed above, different results in microbiota characterization can be attributed mostly to individual and environmenmtal factors, but also to differences in methodologies, population size, reference databases, analytical platforms and assignment tools. *Porphyromonas* is an anaerobic bacterium normally present on the skin and ear of humans and dogs [7,12,21,50]. Although its presence is mostly associated with healthy mucosae, some species have been implicated in respiratory, mucosal, oral and skin infections [51,52]. Our results highlight an increased abundance of *Lactobacillus* in otitis affected group. This bacterium is a common member of the natural microflora associated with gut and skin, including ear [7,19,53], and its presence has been reported as significantly more abundant in atopic dogs when compared to healthy dogs [19]. We rule out that the presence of Lactobacilli is related to previous treatment with probiotics, since the animals included in the present study did not receive such kind of therapy as an alternative treatment of otitis [54], which is a practice that is gaining interest in veterinary practice.

## Conclusions

This study characterized the microbiota of the canine external ear canal through culture-independent methods both in healthy and otitis affected dogs. Our results indicate the presence of a complex bacterial community exhibiting variations both in species richness and distribution not only among different subjects but even within the same animal. Otitis affected dogs show a significant reduction in bacterial variability mostly associated with the proliferation of a single bacterial genus over the others. Although some differences in the bacterial community structure seem to exist in association with specific bacterial pathogens implicated in otitis development, more extensive studies will be necessary to clarify the complex interactions within the ear canal microbial community and between microbiota and host, to contribute to the effective management of the disease.

## Supporting information

S1 Fig Taxa differentially abundant between healthy and affected dogs. ANCOM results identified 17 features as differentially abundant between groups.
(TIF)

S2 Fig Gneiss balance results between healthy and affected dogs. Genera identified by Gneiss as differentially abundant between groups.
(PDF)

S3 Fig Gneiss balance results between sub-set populations of healthy and affected dogs. Genera identified by Gneiss as differentially abundant between groups including only one cerumen sample for each animal (right ear for each animal).
(PDF)

S4 Fig Alpha diversity analysis on a limited sub-set of samples. Alpha diversity analysis carried out on the sub-set of samples including only one cerumen sample for each animal (right ear for each animal).
(TIF)

S5 Fig Beta diversity analysis on a limited sub-set of samples. Beta diversity analysis carried out on the sub-set of samples including only one cerumen sample for each animal (right ear for each animal).
(TIF)

S1 Table Dogs enrolled in the study. Identification number, breed, gender, age, ear and clinical diagnosis for each dog included in the study.
(DOCX)

S2 Table Features found in negative controls. Identification code, abundance and number of negative controls in which each feature has been found.
(XLSX)

S3 Table ANCOM results between healthy and affected dogs. Features identified by ANCOM as differentially abundant between groups. ANCOM analysis carried out on the total population included in the present study.
(XLSX)

S4 Table ANCOM results between healthy and affected dogs. BLAST analysis of the features identified by ANCOM as differentially abundant between groups. ANCOM analysis carried out on the sub-set of population including one cerumen sample for each animal.
(XLSX)

S5 Table Alpha and Beta diversity analysis for variables other than health status. Results from alpha (Kruskal-Wallis test) and beta (PERMANOVA) diversity significance tests for sex and age variables.
(XLSX)
