# Supplementary figures and images for: Cerumen microbial community shifts between healthy and otitis affected dogs
Source: PLoS One. 2020 Nov 25;15(11):e0241447. doi: 10.1371/journal.pone.0241447 (PMC7688138; doi:10.1371/journal.pone.0241447)

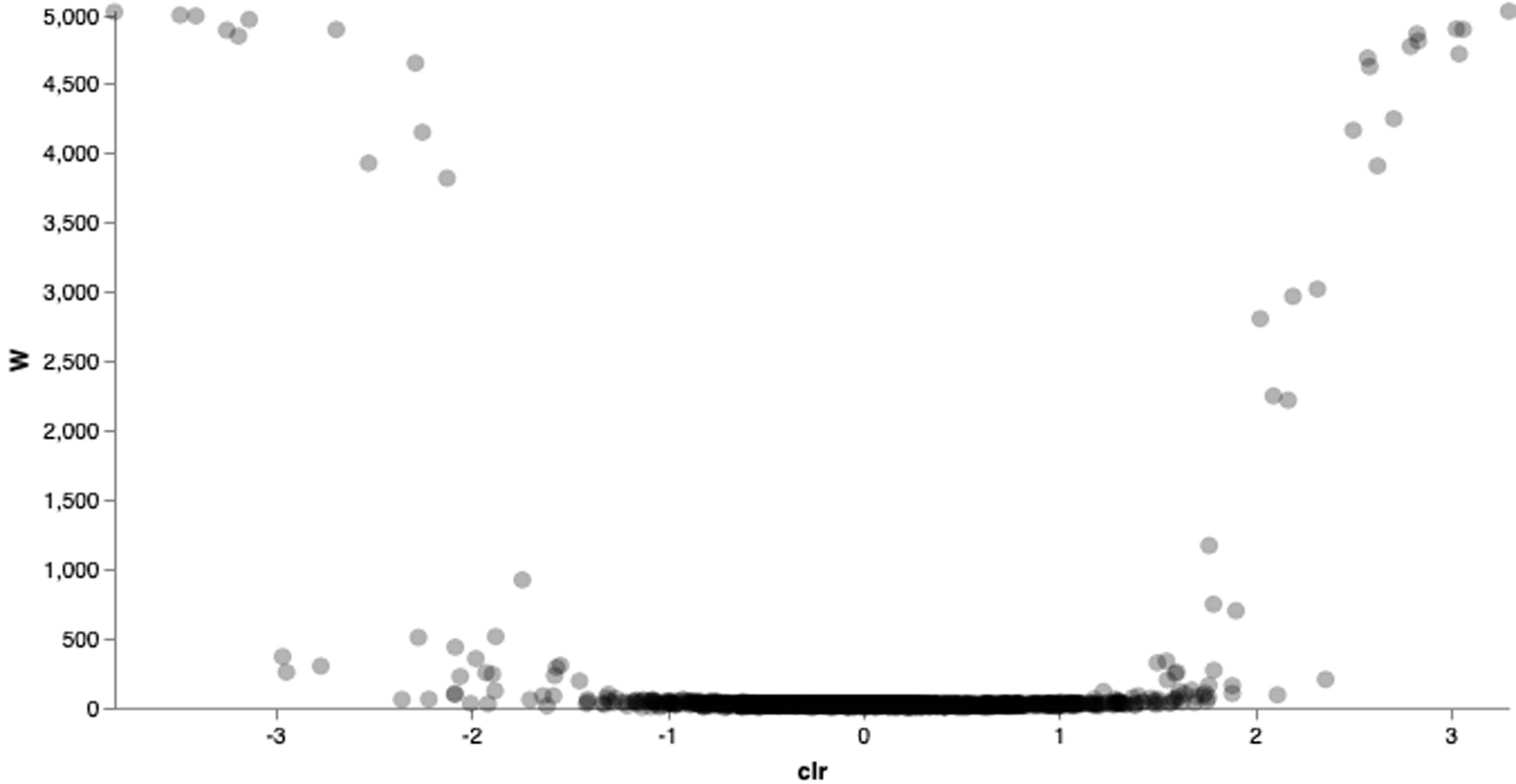

Supplement: S1 Fig — ANCOM results identified 17 features as differentially abundant between groups. (TIF) [file pone.0241447.s001.tif]

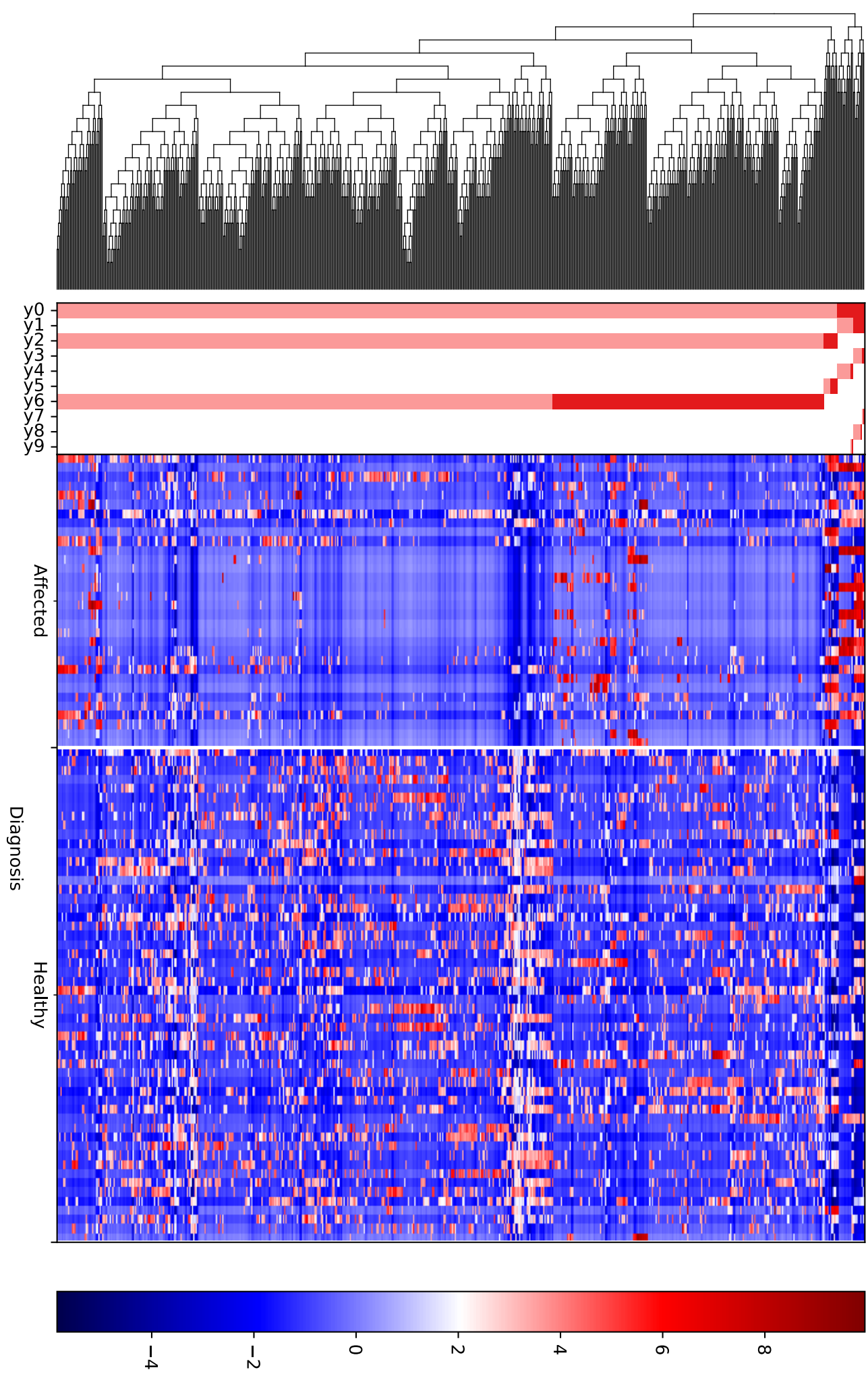

Supplement: S2 Fig — Genera identified by Gneiss as differentially abundant between groups. (PDF) [file pone.0241447.s002.pdf]

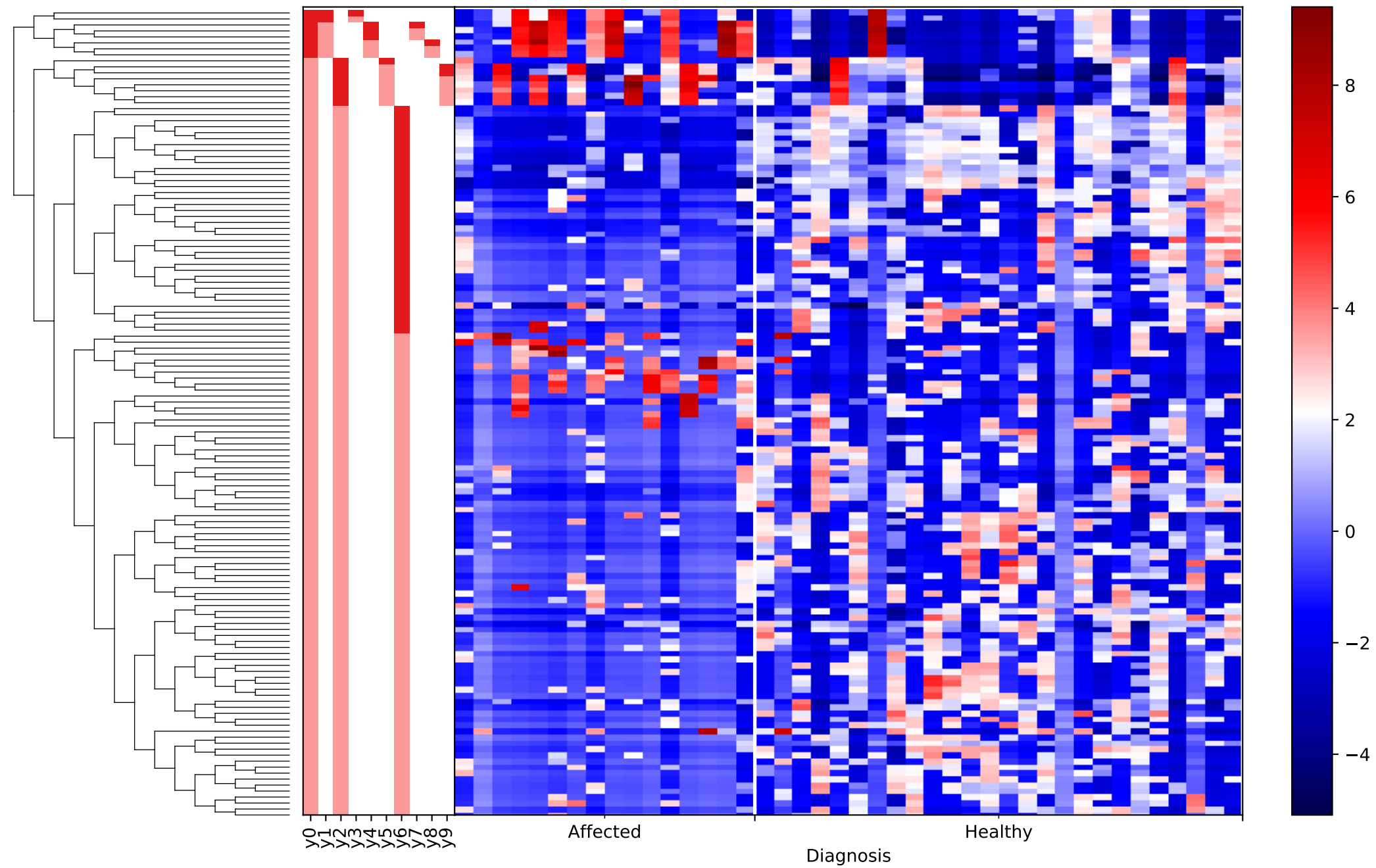

Supplement: S3 Fig — Genera identified by Gneiss as differentially abundant between groups including only one cerumen sample for each animal (right ear for each animal). (PDF) [file pone.0241447.s003.pdf]

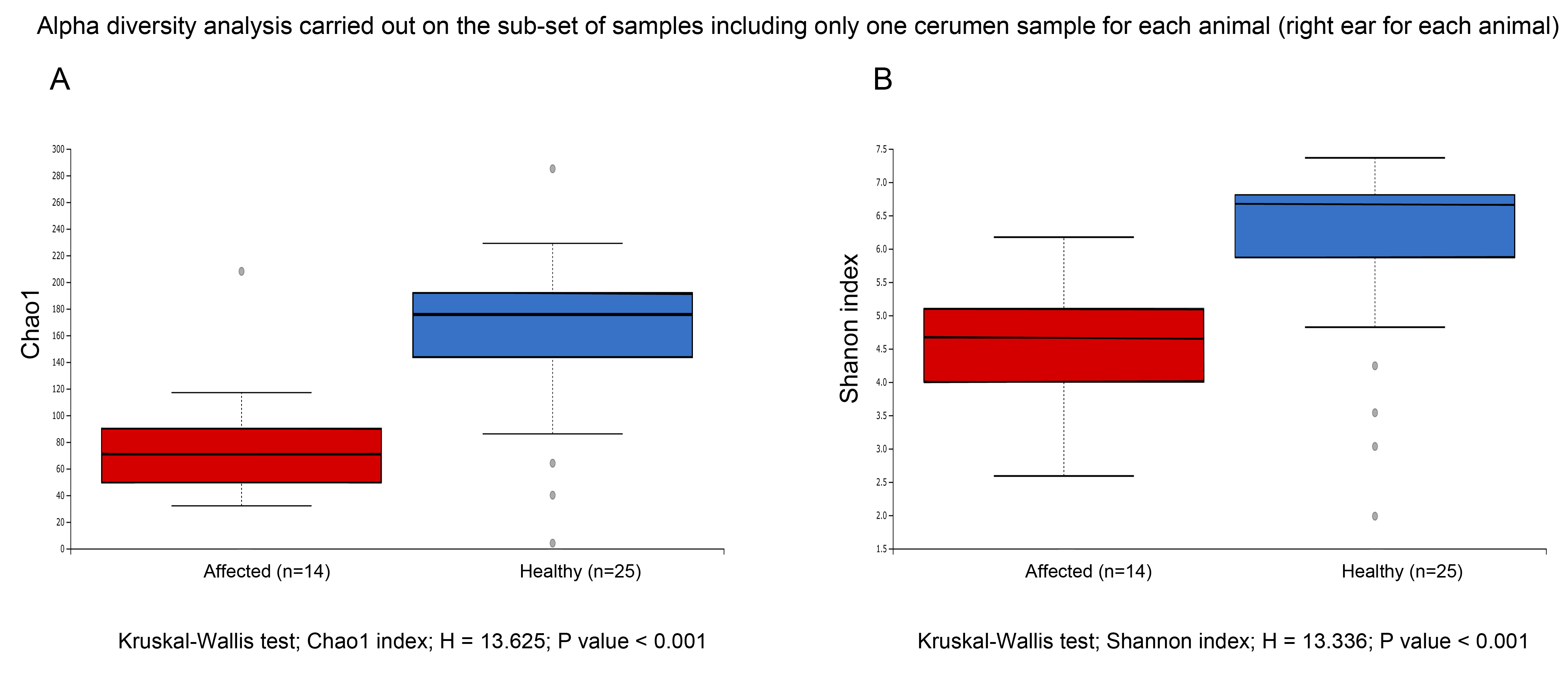

Supplement: S4 Fig — Alpha diversity analysis carried out on the sub-set of samples including only one cerumen sample for each animal (right ear for each animal). (TIF) [file pone.0241447.s004.tif]

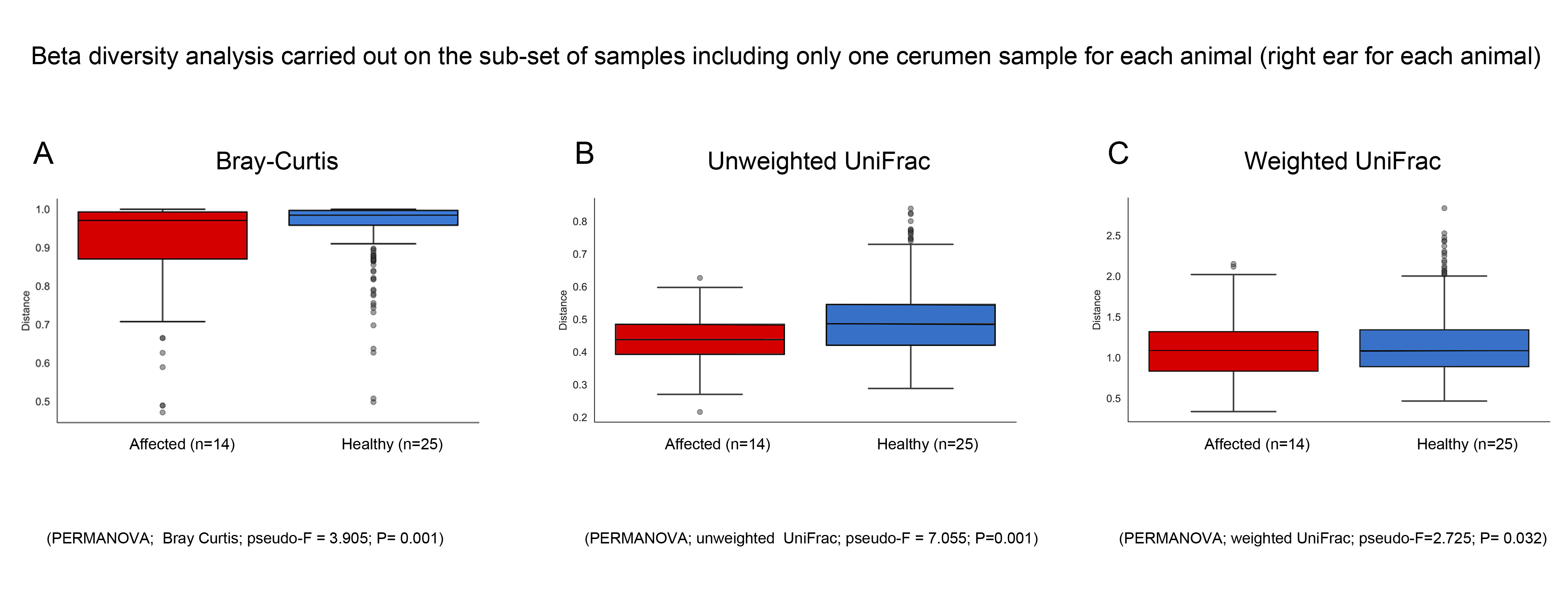

Supplement: S5 Fig — Beta diversity analysis carried out on the sub-set of samples including only one cerumen sample for each animal (right ear for each animal). (TIF) [file pone.0241447.s005.tif]
