# Supplementary material for: Cerumen microbial community shifts between healthy and otitis affected dogs
Source: PLoS One. 2020 Nov 25;15(11):e0241447. doi: 10.1371/journal.pone.0241447 (PMC7688138; doi:10.1371/journal.pone.0241447)
Supplement: S1 Table — Identification number, breed, gender, age, ear and clinical diagnosis for each dog included in the study. (DOCX) [file pone.0241447.s006.docx]

**S1 Table**. **Dogs enrolled in the study**

| ID | Breed | Gender | Age (Years) | Ear | Clinical diagnosis |
| --- | --- | --- | --- | --- | --- |
| 1 | Weimaraner | male | 9 | Right | Healthy |
|  |  |  |  | Left | Healthy |
| 2 | Mixed breed | female | 11 | Right | Healthy |
|  |  |  |  | Left | Healthy |
| 3 | Jack Russell terrier | female | 13 | Right | Healthy |
|  |  |  |  | Left | Healthy |
| 4 | Jack Russel | female | 13 | Right | Healthy |
|  |  |  |  | Left | Healthy |
| 5 | Maltese | male | 2 | Right | Healthy |
|  |  |  |  | Left | Healthy |
| 6 | Mixed breed | male | 3.5 | Right | Healthy |
|  |  |  |  | Left | Healthy |
| 8 | Not reported | female | 10 | Right | Healthy |
|  |  |  |  | Left | Healthy |
| 9 | German shepherd | female | 9 | Right | Healthy |
|  |  |  |  | Left | Healthy |
| 10 | Labrador Hovawart | male | 8 | Right | Healthy |
|  |  |  |  | Left | Healthy |
| 11 | Beagle | male | 10 | Right | Healthy |
|  |  |  |  | Left | Healthy |
| 12 | German shepherd | female | 11 | Right | Healthy |
|  |  |  |  | Left | Healthy |
| 13 | Boxer | female | 7 | Right | Healthy |
|  |  |  |  | Left | Healthy |
| 14 | Not reported | male | 1.5 | Right | Healthy |
|  |  |  |  | Left | Healthy |
| 16 | Cocker | male | 15 | Right | Healthy |
|  |  |  |  | Left | Healthy |
| 18 | Not reported | female | 0.5 | Right | Healthy |
|  |  |  |  | Left | Healthy |
| 19 | Labrador retriever | female | 8 | Right | Healthy |
|  |  |  |  | Left | Healthy |
| 20 | Bouvier des Flandres | female | 11 | Right | Healthy |
|  |  |  |  | Left | Healthy |
| 21 | Not reported | male | 9 | Right | Healthy |
|  |  |  |  | Left | Healthy |
| 22 | Golden retriever | female | 10 | Right | Healthy |
|  |  |  |  | Left | Healthy |
| 23 | Golden retriever | male | 6 | Right | Healthy |
|  |  |  |  | Left | Healthy |
| 24 | Mixed breed | female | 7 | Right | Healthy |
|  |  |  |  | Left | Healthy |
| 25 | Brittany Spaniels | female | 10 | Right | Healthy |
|  |  |  |  | Left | Healthy |
| 27 | Flat- coated retriever | male | 1 | Right | Healthy |
|  |  |  |  | Left | Healthy |
| 28 | Labrador | female | 1 | Right | Healthy |
|  |  |  |  | Left | Healthy |
| 29 | Pitbull | female | 1 | Right | Healthy |
|  |  |  |  | Left | Healthy |
| 30 | French bulldog | Not reported | 1.5 | Right | Healthy |
|  |  |  |  | Left | Healthy |
|  |  |  |  |  |  |
| 7 | Rhodesian Ridgeback | male | 1.5 | Right | Otitis |
|  |  |  |  | Left | Healthy |
| 34 | Labrador | male | 9 | Right | Healthy |
|  |  |  |  | Left | Otitis |
| 35 | Mixed breed | female | 10 | Right | Otitis |
|  |  |  |  | Left | Healthy |
| 40 | Mixed breed | male | 8 | Right | Healthy |
|  |  |  |  | Left | Otitis |
|  |  |  |  |  |  |
| 15 | Cocker | male | 14 | Right | Otitis |
|  |  |  |  | Left | Otitis |
| 17 | Not reported | male | 6 | Right | Otitis |
|  |  |  |  | Left | Otitis |
| 26 | Dalmatian | female | 8 | Right | Otitis |
|  |  |  |  | Left | Otitis |
| 36 | Mixed breed | female | 8,5 | Right | Otitis |
|  |  |  |  | Left | Otitis |
| 37 | French bulldog | female | 1 | Right | Otitis |
|  |  |  |  | Left | Otitis |
| 38 | Labrador | male | 2 | Right | Otitis |
|  |  |  |  | Left | Otitis |
| 39 | German shepherd | female | 3 | Right | Otitis |
|  |  |  |  | Left | Otitis |
| 41 | West Highland White Terrier | Not reported | 7 | Right | Otitis |
|  |  |  |  | Left | Otitis |
| 42 | German shepherd | female | 11 | Right | Otitis |
|  |  |  |  | Left | Otitis |
| 43 | Labrador |  | 9 | Right | Otitis |
|  |  |  |  | Left | Otitis |
| 44 | English bulldog | male | 3 | Right | Otitis |
|  |  |  |  | Left | Otitis |
| 45 | Cocker | male | 8 | Right | Otitis |
|  |  |  |  | Left | Otitis |
| 46 | German shepherd | female | 9 | Right | Otitis |
|  |  |  |  | Left | Otitis |
| 47 | English bulldog | male | 11 | Right | Otitis |
|  |  |  |  | Left | Otitis |
| 48 | Labrador retriever | female | 1 | Right | Otitis |
|  |  |  |  | Left | Otitis |
| 49 | Labrador | male | 2 | Right | Otitis |
|  |  |  |  | Left | Otitis |
